# Synthesis, characterization, anti-proliferative, and apoptotic activity of a novel quinazoline-containing 1,2,3-triazole toward three human cancer cells

**DOI:** 10.1038/s41598-025-05270-z

**Published:** 2025-07-01

**Authors:** Mohammad Javad Dehghan-Nayeri, Fariba Peytam, Alireza Foroumadi, Majid Mahdavi

**Affiliations:** 1 Department of Biology, Faculty of Natural Sciences, University of Tabriz, Tabriz, Iran; 2 Drug Design and Development Research Center, The Institute of Pharmaceutical Sciences (TIPS), Tehran University of Medical Sciences, Tehran, Iran; 3 Department of Medicinal Chemistry, Faculty of Pharmacy, Tehran University of Medical Sciences, Tehran, Iran; 4 Institute of Biochemistry and Biophysics, University of Tehran, Tehran, Iran

**Keywords:** Quinazoline, Apoptosis, Cancer cell, VEGFR2, Biochemistry, Cancer, Cell biology, Chemical biology, Drug discovery

## Abstract

A novel quinazoline-containing 1,2,3-triazole **(4-TCPA)** was synthesized to target VEGFR2 signaling for cancer treatment. Although current VEGFR2 inhibitors exhibit strong anticancer activity, their clinical use is limited due to severe side effects. Structural analysis of effective VEGFR2 inhibitors guided the design of **4-TCPA**, ensuring the retention of Erlotinib’s essential pharmacophoric features for optimal receptor binding. The compound was tested for its anticancer efficacy against A549 (lung cancer), MCF7 (breast cancer), K562 (leukemia), and human normal fibroblast HFF2 cells. **4-TCPA** demonstrated inhibitory activity in nearly all assays, with IC_50_ concentrations of 35.70 μM for A549, 19.50 μM for MCF7, 5.95 μM for K562, and 135.2 μM for HFF2. Further in vitro biological evaluations examined its mechanistic impact on EGFR, mTOR, Akt, MAPK, and PIK3CA. Apoptotic activity was assessed through Annexin V/PI double staining and caspase-3/7 activation, confirming its ability to induce both early and late apoptosis. Additionally, real-time quantitative PCR (qRT-PCR) was conducted to evaluate the expression levels of Akt, mTOR, MAPK, PIK3CA, EGFR, and VEGFR2. Results showed that **4-TCPA** down-regulates these targets in a time-dependent manner, triggering apoptosis across all tested cancer cell lines. The study suggests that **4-TCPA** may serve as a promising anticancer agent due to its targeted VEGFR2 inhibition and apoptotic induction, warranting further investigation. These findings support the potential of **4-TCPA** as an effective treatment strategy for various cancers.

**Supplementary Information:**

The online version contains supplementary material available at 10.1038/s41598-025-05270-z.

## Introduction

The second most common cause of mortality in the world is cancer. Even with the quick advancements in diagnostic techniques and treatment approaches, the primary barriers to effective treatment are metastasis and drug resistance. Therefore, diagnosing and developing the best therapeutic approaches for patients should be the primary focus to maximize their chances of responding well to treatment and extending their life. It has been discovered that several pathways are dysregulated in many forms of cancer. This covers anomalies in the cell cycle, apoptosis, angiogenesis, metabolic processes within cells, or epigenetic changes. Furthermore, signals from the microenvironment may play a significant role in developing cancer^1^. Therefore, one of the main challenges scientists’ faces is developing novel, potent anti-cancer medications. Novel anti-cancer therapies have demonstrated efficacy in both in vitro and clinical trials; therefore, there is a good chance, but no assurance, that these medications will work in vivo^2^. Multidrug resistance could result from medication use and could cause significant challenges in treating cancer. Furthermore, a growing body of research suggests that elements in the microenvironment could affect a cell’s reaction to treatment^3^. Numerous cellular processes, including proliferation, differentiation, and neoplastic transformation, are linked to phosphatases and kinases. These proteins are critical in modulating intracellular signaling pathways facilitating various cellular processes^4^. The nonreceptor tyrosine kinases (nRTKs) and receptor tyrosine kinases (RTKs) proteins comprise the family of protein tyrosine kinases. In addition to controlling several biological functions in healthy cells, RTKs have been linked to the onset and spread of cancer. Indeed, constitutive receptor activation and uncontrollably activating several signal transduction pathways are caused by mutations in RTKs^5^. There have been descriptions of over 20 distinct RTK classes, including EGFR, insulin R, PDGFR, VEGFR, FGFR, HGFR, and RET^4^. Creating new blood vessels from preexisting microvascular structures is known as angiogenesis. The onset of this process is characterized by the activation of endothelial cells, dilatation of the vascular basement membrane, and an increase in interstitial matrix permeability. Endothelial cell migration, survival, and proliferation critically depend on this biological signaling. Numerous stimulating and inhibiting variables tightly govern normal physiological angiogenesis. Proangiogenic factors, in particular, balance antiangiogenic factors, such as TSP, angiostatin, and endostatin; these include growth factors, including VEGF, FGF, PDGF, HGF, and EGF; adhesion factors; proteinases; extracellular matrix proteins; transcription molecules; and signaling factors^6,7^. It has been documented that cancer cells are resistant to chemotherapy, proliferate, and survive due to abnormal vascular endothelial growth factor (VEGF) signaling^8^. It has been previously shown that 1,2,3-triazole, one of the isomeric forms of triazoles, exhibits intense anti-cancer action in various malignancies. Many attempts have been made over the past 20 years to create 1,2,3-triazoles, which induce apoptosis, and VEGFR-2 inhibitors^9^. Many small molecules of VEGFR-2 inhibitors, including Pazopanib, Sunitinib, Sorafenib, and Axitinib, have been created and authorized as effective anti-cancer medications. Clinical experience indicates that these medicines are less likely to develop resistance and have broad-spectrum anti-tumor activity. However, the side effects that result in stopping treatment and reducing dosage may outweigh the ability of these medications to prolong life, highlighting the unmet need for more effective and safer VEGFR-2 inhibitors^10^. Heterocyclic rings containing nitrogen and sulfur are of significant intention as they are therapeutically and pharmacologically active. These chemicals are the building components of numerous medicinal medicines. Quinazoline has been selected for this review out of all heterocyclic moieties since it has a wide range of pharmacological activity and few adverse effects. Quinazoline, which has the chemical formula C8H6N2, is a well-known heterocyclic compound. *Quinazoline* is a light-yellow crystalline compound composed of one pyrimidine and one benzene ring. It is often referred to as 1,3-diazanaphthalene, and by decarboxylating a 2-carboxy derivative, August Bischler, and Lang reported synthesizing quinazoline in 1895. Niementowski synthesis was used to produce 4-oxo-3,4-dihydroquinazolies from anthranilic acid treated with amide. Other isomers of quinazoline include phthalizine, cinnoline, and quinoaxoline. Additionally, quinazolines are the building blocks of more than 200 naturally occurring alkaloids in animals, microorganisms, and plants. The first quinazoline alkaloid, vasicine ( ±), also called peganine, was discovered in Adhatoda vasica in 1888. Bronchodilator activity works quite well^11,12^. Quinazoline derivatives are thought to be highly effective chemotherapeutic treatments for solid tumors. Many commercially available medications are built on the quinazoline/quionazolinone scaffold. Non-small cell lung cancer can be treated with Gefitinib, Erlotinib, and Afatinib; breast cancer can be treated with Lapatinib; advanced medullary thyroid carcinoma can be treated with Vandetanib (Caprelsa®); advanced colorectal cancer can be treated with Raltitrexed (Tomudex®); and various cancers, including colorectal, breast, renal, and lung cancer, can be treated with Cediranib (Recentin®)^13–15^ (Table 1). The emergence of targeted therapies has revolutionized cancer treatment, yet the challenge of drug resistance remains a significant hurdle. Recent studies have highlighted the role of the tumor microenvironment in mediating resistance to therapies, emphasizing the need for combination strategies that can effectively target multiple pathways^16^. Furthermore, identifying of novel biomarkers for patient stratification is crucial for optimizing treatment regimens and improving clinical outcomes^17^. Research indicates that the interaction between cancer cells and surrounding stromal cells can significantly affect the efficacy of therapies targeting RTKs. For instance, a study by Frisbie et al.^18^ demonstrated that modulating the tumor microenvironment can enhance the sensitivity of cancer cells to VEGFR2 inhibitors. In this context, the development of quinazoline-based compounds has garnered attention due to their ability to inhibit key signaling pathways involved in tumor growth and metastasis^19^. For instance, recent findings suggest that compounds targeting the VEGFR2 pathway can significantly impair angiogenesis and tumor progression^20^. These advancements underscore the importance of continued research into the structural modifications of quinazoline derivatives to enhance their therapeutic efficacy^21^. In addition to targeting kinase and phosphatase signaling, developing multi-targeted agents simultaneously inhibiting multiple oncogenic pathways has recently gained significant attention. Combination therapies that target both angiogenesis and other key signaling cascades, such as EGFR and mTOR, have demonstrated promising results in preclinical and clinical studies. The ability of quinazoline-containing 1,2,3-triazole compounds to modulate the expression of genes involved in VEGFR-2, EGFR, and mTOR signaling suggests that these novel agents may have the potential to overcome the limitations of existing VEGFR-2 inhibitors and provide a more comprehensive approach to cancer treatment^22^. In addition to targeting RTKs, developing of small molecule inhibitors that can selectively modulate downstream signaling pathways is gaining traction. A study by Lin et al.^23^ reported the synthesis of novel compounds that inhibit the PI3K/Akt pathway, which is often dysregulated in cancer. These compounds showed promising results in preclinical models, suggesting a new avenue for enhancing the therapeutic efficacy of existing treatments. Moreover, integrating novel therapeutic agents with existing treatment modalities, such as immunotherapy and chemotherapy, presents a promising avenue for overcoming resistance. Combining targeted agents with immunotherapeutic approaches has shown synergistic effects in preclinical models, paving the way for innovative treatment strategies^24^. Furthermore, integrating artificial intelligence and machine learning in drug discovery is revolutionizing the identification of potential anti-cancer agents. Recent advancements in computational methods have enabled researchers to predict the activity of novel quinazoline derivatives more accurately. According to a study by Dhuguru and Ghoneim^25^, AI-driven approaches have accelerated the discovery of compounds with improved selectivity and reduced toxicity profiles. Lastly, ongoing clinical trials are crucial for validating the safety and efficacy of new therapeutic agents. The results from recent trials involving novel VEGFR2 inhibitors, such as those reported by Cho et al.^26^, indicate that these agents exhibit enhanced anti-tumor activity and demonstrate a favorable safety profile compared to traditional therapies. This underscores the importance of continued research to bring innovative treatments to patients.

**Table 1:** The FDA approved VEGFR & EGFR inhibitors.

Compound	Chemical structure	Primary target(s)	IC_50_ value(s)	Reference
Pazopanib		VEGFR1, VEGFR2, VEGFR3, PDGFRα/β, c-Kit	VEGFR2: 30 nM	PubChem
Sunitinib		VEGFR2, PDGFRβ, c-Kit	VEGFR2: 80 nM	PubChem
Sorafenib		VEGFR2, VEGFR3, PDGFRβ, FLT3, c-Kit	VEGFR2: 90 nM	PubChem
Axitinib		VEGFR1, VEGFR2, VEGFR3, PDGFRβ, c-Kit	VEGFR2: 0.2 nM	PubChem
Erlotinib		EGFR	EGFR: 2 nM	PubChem
Gefitinib		EGFR	EGFR: 33 nM	PubChem
Afatinib		EGFR, HER2	EGFR: 0.5 nM	PubChem
Vandetanib		VEGFR2, VEGFR3, EGFR	VEGFR2: 40 nM	PubChem
Raltitrexed		Thymidylate Synthase	TS: 9 nM	PubChem
Cediranib		VEGFR2, VEGFR1, VEGFR3, PDGFRα/β, c-Kit	VEGFR2: <1 nM	PubChem

In this study, we synthesized a novel quinazoline-containing 1,2,3-triazole compound **(4-TCPA)** to investigate its anti-cancer potential, particularly as inhibitors of VEGFR2. This is significant as VEGFR2 plays a crucial role in tumor angiogenesis, supporting cancer cells’ growth and metastasis. By designing and developing this novel compound, we aimed to evaluate its efficacy in inhibiting cancer cell proliferation and inducing apoptosis across different cancer cell lines, including A549 (lung cancer), MCF7 (breast cancer), and K562 (leukemia), while also examining their selectivity by testing on normal human fibroblast cells (HFF2). Our approach was grounded in medicinal chemistry principles, leveraging the structural pharmacophore features of Erlotinib, a well-known kinase inhibitor, to enhance receptor binding affinity and therapeutic potential.

## Material and methods

### Materials

The cell culture medium (DMEM) and fetal bovine serum (FBS) were purchased from Thermo Fisher Scientific Inc (Biotechnology company, USA). The supplier of penicillin–streptomycin was Gibco (Life Technologies, USA). Sigma Aldrich (St. Louis, Missouri, USA) supplied the 3-(4,5-dimethyl-2-thiazolyl)-2, 5-diphenyl-2-H-tetrazolium bromide (MTT), Erlotinib, and Propidium Iodide (PI). We purchased the Caspase-3 and 7 assay kit from BD Biosciences Pharmingen in San Diego, California, USA. Merck, a German company, supplied the dimethyl sulfoxide (DMSO). Sina Clon (Iran) provided the RNA isolation kit. The Prime Script™ RT reagent kit (Takara, Japan) was used for cDNA synthesis and Real-Time PCR, while AmpliQon (AmpliQon, Denmark) supplied the RealQ Plus 2 × Master Mix Green High ROX™. EGFR, VEGFR2, mTOR, Akt, MAPK, and PIK3CA were primers acquired from Santa Cruz Biotechnology Inc. (Santa Cruz, CA, USA). All cell lines were obtained from the Pasteur Institute of Iran (Tehran).

### The general procedure for the preparation of the novel compound

Merck (Germany) supplied all compounds, which were utilized without additional purification. Thin-layer chromatography (TLC) on silica gel 250-micron F254 plastic sheets was used to track the reaction’s progress and the purity of the produced chemicals; zones were visually identified under UV light (254 nm). The Bruker DRX-500 AVANCE instrument was used to measure the ^1^H and ^13^C NMR spectra (in DMSO-*d*_*6*_ solution) at 500.1 and 125.8 MHz. Downfield chemical changes from tetramethylsilane were recorded in parts per million (ppm). Singlet (s), doublet (d), triplet (t), and multiplet (m) were the terms used to characterize proton coupling patterns. High-resolution mass spectrometry (HRMS) analysis was performed using a Waters Synapt G1 HDMS High Definition mass spectrometer equipped with an electrospray ionization (ESI) source. The samples were prepared by diluting the isolated compounds in methanol to a final concentration of 10 µg/mL. The analysis was conducted mainly in positive ion mode with a mass range of m/z 50–1000.

### General procedure for the preparation of 6,7-bis(2-methoxyethoxy)-*N*-(prop-2-yn-1-yl) quinazolin-4-amine 8

To a stirring solution of propargyl amine **7** (2 mmol, 0.106 g) and Et_3_N (2 mmol, 0.28 mL) in DMF (10 mL) at 80 °C, 4-chloro-6,7-bis(2-methoxyethoxy) quinazoline **6** (1.538 mmol, 0.481 g) was added gradually and heated for 12 h. Upon completion of the reaction as confirmed by TLC, the reaction mixture was cooled to the ambient temperature. Subsequently, water (50 mL) was added to the mixture and extracted three times with EtOAc (3 × 50 mL). The combined organic extracts were washed with brine, dried over sodium sulfate (Na_2_SO_4_), and then concentrated. Finally, the residue was recrystallized from ethanol, affording the pure desired compound **8** as a brown solid in 65%.

### General procedure for the preparation of 2-(4-(((6,7-bis(2-methoxyethoxy) quinazolin-4-yl) amino) methyl)-1*H*-1,2,3-triazol-1-yl)-*N*-(4-chlorophenyl) acetamide 9 (4-TCPA)

A mixture of 2-azido-*N*-(4-chlorophenyl)acetamide **5** (1 mmol, 0.212 g, which was synthesized using our previously reported method^27^), 6,7-bis(2-methoxyethoxy)-*N*-(prop-2-yn-1-yl) quinazolin-4-amine** 8** (1 mmol, 0.331 g), CuSO_4_·5H_2_O (0.3 mmol, 0.075 g), and sodium ascorbate (0.3 mmol, 0.059 g) in DMF (5 mL) was stirred at room temperature within overnight. Water (25 mL) was then added to the reaction mixture, and stirring was continued until a precipitate formed completely. The resulting solid was collected by filtration and thoroughly washed with water. Finally, the product was recrystallized from ethanol, yielding the pure targeted compound, 2-(4-(((6,7-bis(2-methoxyethoxy) quinazolin-4-yl) amino) methyl)-1*H*-1,2,3-triazol-1-yl)-*N*-(4-chlorophenyl) acetamide **9** (**4-TCPA**), as a milky powder with a 73% yield.

Milky solid, mp 212–214 °C, yield: 73%. ^1^H NMR (500.1 MHz, DMSO-*d*_6_): *δ* 10.58 (s, 1H, NH), 8.59 (s, 1H, CH), 8.15 (s, 1H, CH), 7.87 (br. s, 1H, NH), 7.60 (d, *J* = 8.5 Hz, 2H, 2CH), 7.36 (d, *J* = 8.5 Hz, 2H, 2CH), 7.34 (s, 1H, CH), 7.20 (s, 1H, CH), 5.32 (s, 2H, CH_2_N), 4.73 (d, *J* = 4.6 Hz, 1H, CH_2_NH), 4.35–4.20 (m, 4H, 2CH_2_O), 3.80–3.70 (m, 4H, 2CH_2_O), 3.35 (s, 6H, 2OCH_3_). ^13^C NMR (126 MHz, DMSO-*d*_6_) *δ* 165.91, 164.82, 155.45, 152.07, 149.69, 137.81, 134.05, 130.29, 129.27, 127.80, 125.53, 121.21, 118.75, 107.13, 102.74, 70.52, 68.83, 58.81, 52.66, 45.67. HRMS (ESI) m/z for C_25_H_29_ClN_7_O_5_^+^ [M + H]^+^, calculated: 542.1913, found: 542.1912.

### Cell culture

In DMEM medium containing 10% fetal bovine serum (FBS), 100 units/mL of penicillin, and 100 μg/mL of streptomycin, the human lung cancer (A549), human chronic myeloid leukemia (CML) K562, human breast cancer (MCF7), and normal human foreskin fibroblast HFF2 cell lines were cultured at 37 °C in a humidified atmosphere with 5% CO_2_.

### MTT assay

Target compounds were tested for their cytotoxic potential at specific doses against several human cancer cell lines (A549, K562, MCF7, and HFF2). To enable cell adhesion, 10,000 viable cells were seeded into each well of 96-well plates, and the plates were then incubated for an entire night. After that, they were cultured at 37 °C for 48 h while being exposed to different doses of previously synthesized chemicals (in DMSO). Following the PBS rinse, each well-received 20 μl of 3-(4,5-Dimethyl-2-thiazolyl)-2,5-diphenyl-2H-tetrazolium bromide (MTT) solution (0.5 mg/mL). After an additional 4 h of incubation at 37 °C, the media was discarded. In this stage, the reductase enzyme assisted in the transformation of MTT into purple tetrazolium crystals. The inclusion of DMSO (100 μl) as a suitable solvent in each well significantly improved the solubility of these purple crystals^28^. At a test wavelength of 570 nm, the absorbance was measured using a plate reader (Bio-Rad microplate reader, Model 680). The percentage of living cells is precisely proportional to the intensity of absorbance measured by the plate reader. To quantitatively assess whether or not the finished compounds are cytotoxic, a very basic and imprecise estimation approach called half maximum inhibitory concentration (IC_50_) was used in the numerical computation.

### Apoptosis determination using Annexin V-FITC/PI

Using flow cytometry (FCM) to detect apoptosis, the apoptosis detection kit from BD Biosciences Pharmingen (San Diego, CA, USA) was utilized as directed by the manufacturer. Cells (8 × 10^5^ cells) were treated with IC_50_ for 72 h in a 12-well plate at 37 °C and 5% humidified CO_2_. After being collected, the cells were washed with cold PBS and suspended in a binding buffer. Tags: Annexin V, PI. A 30-min dark staining process was conducted at 4 °C. Cells were examined using the FACS Calibur flow cytometer from Becton Dickinson, Franklin Lakes, New Jersey, USA, and quantified using FlowJo v10 (Becton, Dickinson and Company, Franklin Lakes, NJ, USA)^29^.

### Caspase 3/7 like activity assay

Compound 12 was used to treat cancer cells for 72 h at its IC_50_ concentration. Following the manufacturer’s instructions, the cells were then harvested, and Caspase 3/7 activity was determined using a colorimetric activity assay kit (Caspase 3/7 Assay Kit, Colorimetric, BD Biosciences, USA) (Biosciences BD Biosciences-US, 2020). The chromogenic substrate cleavage, Ac-DEVD-pNA, following caspase 3/7 activation is used in the analysis^30^. A microplate reader (Bio-TEK, USA) measured the released pNA moiety, which correlates directly with caspase 3/7 activities at 405 nm.

### Gene expression analysis

Following a 72-h drug treatment, the total RNA from both treated and untreated cancer cells was extracted using the RNX-plus reagent (Cinnagen, Iran) by the manufacturer’s advice. 1% agarose gel electrophoresis and Nanodrop (Merck, Germany) were used to assess the quantity and quality of isolated RNAs, respectively. Using the Prime Script RT reagent kit (Takara Bio, USA) and the manufacturer’s instructions, 1 μg of total RNA was reverse transcribed to synthesize complementary DNA (cDNA). qRT-PCRs were performed with RealQ Plus 2 × Master Mix Green High ROXTM (Amplicon, Denmark) on Step 1 software. The expression levels of Akt, mTOR, MAPK, PIK3CA, EGFR, and VEGFR2 genes were compared to the untreated cells (control) and were normalized against the housekeeping gene, Beta-2-Microglobulin (B2M). The comparative ΔΔCt technique was used to analyze each mRNA molecule’s expression level. Table 2 displays the sequences of the qPCR primers that Oligo (Macrogen, Seoul, South Korea) manufactured. The qPCR methodology was as follows: a single denaturation cycle at 95 °C for 5 min was followed by forty amplification cycles at 95 °C for 30 s, 60–64 °C for 30 s, 72 °C for 30 s, and a final extension at 72 °C for 5 min. A melting curve analysis was performed on each individual run to assess primer dimer absence and amplification specificity. Lastly, LinReg PCR 2017.9 version was used to calculate the mean amplification efficiencies for each primer. Every experiment was run three times.

**Table 2:** Primers sequences used in reverse transcription real-time quantitative polymerase chain reactions.

Gene	Primer	Sequences	Amplicon (bp)
B2M	Forward	5′-GACCACTTACGTTCATTGACTCC-3′	170
	Reverse	5′-CAGGGTTTCATCATACAGCCAT-3′	
Akt	Forward	5′-ACCTGACCAAGATGACAGC-3′	160
	Reverse	5′-ATACAGATCATGGCACGAGG-3′	
mTOR	Forward	5′-GAACAGTGAGCACAAGGAG-3′	180
	Reverse	5′-ATGTCAGGGTCAGGATCTG-3′	
MAPK	Forward	5′-ACACCAACCTCTCGTACATCGG-3′	124
	Reverse	5′-TGGCAGTAGGTCTGGTGCTCAA-3′	
PIK3CA	Forward	5′-GTACCTTGTTCCAATCCCAG-3′	137
	Reverse	5′-GGACAGTGTTCCTCTTTAGC-3′	
EGFR	Forward	5′-AACACCCTGGTCTGGAAGTACG-3′	106
	Reverse	5′-TCGTTGGACAGCCTTCAAGACC-3′	
VEGFR2	Forward	5′-ATCCCAGATGACAACCAGAC-3′	138
	Reverse	5′-TTCAGATGCCACAGACTCC-3′	

### Statistical analysis

The mean ± SD is used to illustrate the triplicate quality of the experiments. Data were analyzed using GraphPad Prism software, version 9.0.0 (GraphPad Software, CA, USA), either with an independent sample t-test or a one-way ANOVA followed by Tukey’s Post-hoc test. The significance level of the results was set at *p* values < 0.05(*), < 0.01 (**), < 0.001 (***), and < 0.0001 (****).

## Results

### Synthesis, characterization and determination of the chemical structure

**Fig. 1:**
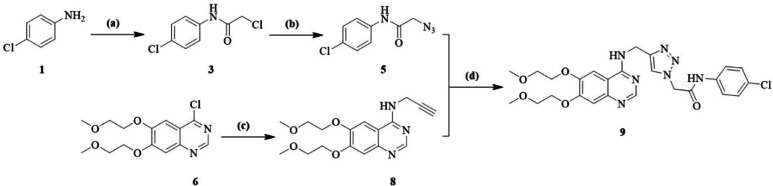
Reaction conditions and reagents: (**a**) chloroacetyl chloride **2** (1.2 equiv.), Et_3_N (1.2 equiv.), acetone, r.t., overnight; (**b**) NaN_3_ **4** (1.5 equiv.), DMF, r.t., overnight; (**c**) propargyl amine **7** (1.3 equiv.), Et_3_N (1.3 equiv.), DMF, 80 °C, 12h; (**d**) CuSO_4_.5H_2_O (0.3 equiv.), sodium ascorbate (0.3 equiv.), DMF, r.t., overnight.

2-(4-(((6,7-bis(2-methoxyethoxy) quinazolin-4-yl) amino) methyl)-1*H*-1,2,3-triazol-1-yl)-*N*-(4-chlorophenyl) acetamide **9 (4-TCPA)** was prepared through an efficient, multi-step synthetic route, as shown in Fig. 1. Initially, 4-chloro-6,7-bis(2-methoxyethoxy) quinazoline **6** went through the nucleophilic aromatic substitution (S_N_Ar) with propargyl amine **7** in the presence of triethylamine (Et_3_N), producing 6,7-bis(2-methoxyethoxy)-*N*-(prop-2-yn-1-yl) quinazolin-4-amine **8**. On the other hand, 2-azido-*N*-(4-chlorophenyl)acetamide **5** was prepared using the procedure reported in our previous work^27^. Finally, this compound was subjected to a click reaction with the acetylene derivative **8** in the presence of CuSO_4_.5H_2_O and sodium ascorbate in DMF at room temperature for an overnight, leading to the formation of the targeted compound **9 (4-TCPA)**. The structure of this compound was confirmed using ^1^H-NMR (Fig. S1), ^13^C-NMR (Fig. S2) spectroscopy, as well as high-resolution mass spectrometry (HRMS) (Fig. S3).

### Effects of the quinazoline containing 1,2,3-triazole derivatives on cells viability

**Fig. 2:**
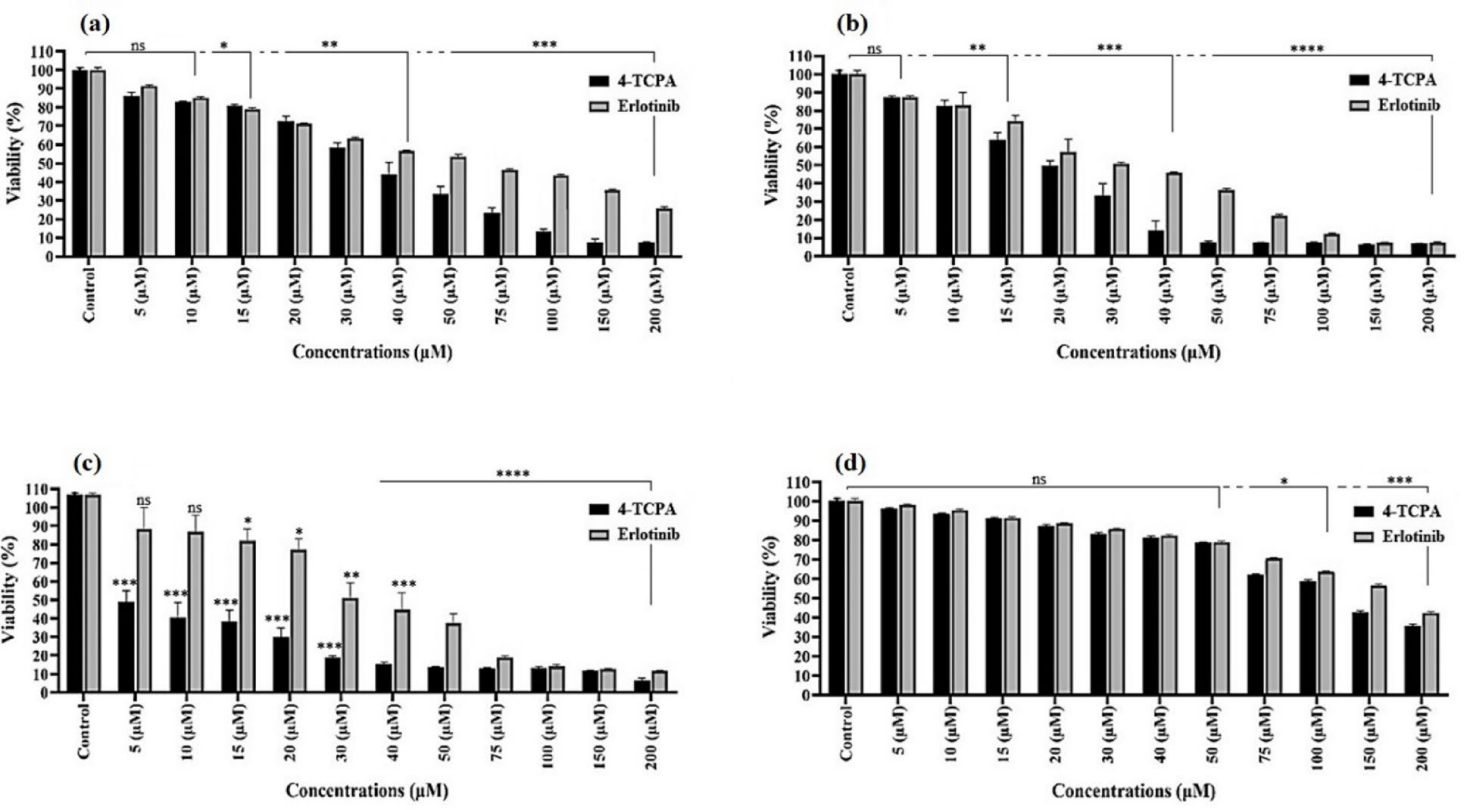
The cytotoxicity induction of **4-TCPA** and Erlotinib (as a positive control) on A549 (**a**), MCF7 (**b**), K562 (**c**) and HFF2 (**d**) cell lines. The chemical was applied to the cells at different concentrations (0–200 µg/mL) during 72 h. The data reflect the mean plus standard deviation (SD) of three separate studies. By contrasting each group with its control, values of *p < 0.05, **p < 0.01, ***p < 0.001 and ****p < 0.0001 were discovered.

In order to determine the impact of quinazoline containing 1,2,3-triazole derivative **(4-TCPA)** on the proliferation and viability of cancer cells, 1 × 10^4^ cells/well were subjected to varying concentrations (0–200 μM) of the derivative for 72 h, and the colorimetric MTT test was employed to detect cytotoxicity. The outcome demonstrated a dose- and time-dependent decrease in the viability and proliferation of cell lines. Table 3 compiled the compounds’ IC_50_ concentrations after a 72-h exposure. The synthetic compound was tested for its ability to inhibit the growth of different cancer cell lines, and the results were presented as IC_50_ concentrations. The related cancer cell lines were purposefully chosen from a variety of cancer sources, such as A549 (lung cancer), MCF7 (breast cancer), K562 (leukemia), and normal human foreskin fibroblast cells (HFF2). **4-TCPA** had the potency, with an IC_50_ concentration of 35.70 μM, 19.50 μM, 5.95 μM, and 135.2 μM against A549, MCF7, K562, and HFF2, respectively (Fig. 2a–d). It was shown that **4-TCPA** exhibited nearly twofold more activity in producing cytotoxic effects on the cancer cell line when compared to Erlotinib. In addition, normal human foreskin fibroblast cell (HFF2) was used in the MTT assay to assess the selectivity of the active compound on cancer cells. The chemical showed no discernible action when comparing the cancer cells to the normal HFF2 cell line. **4-TCPA** was somewhat hazardous to the regular HFF2 cell line. Generally speaking, 1,2,3-triazole contributed to the rise in anti-proliferative activity against cancer cell lines.

**Table 3:** The IC_50_ values of quinazoline containing 1,2,3-triazole derivative (4-TCPA) after 72 h of exposure in A549, MCF7, K562 and HFF2 cell lines.

Compound name	A549	MCF7	K562	HFF2
4-TCPA	35.70 µM	19.50 µM	5.95 µM	135.2 µM
Erlotinib	70.18 µM	36.73 µM	37.33 µM	191.0 µM

Each value represents the mean of three independent experiments ± S.D. (*p* < 0.0001).

### Assessment of apoptosis by flow cytometry

**Fig. 3:**
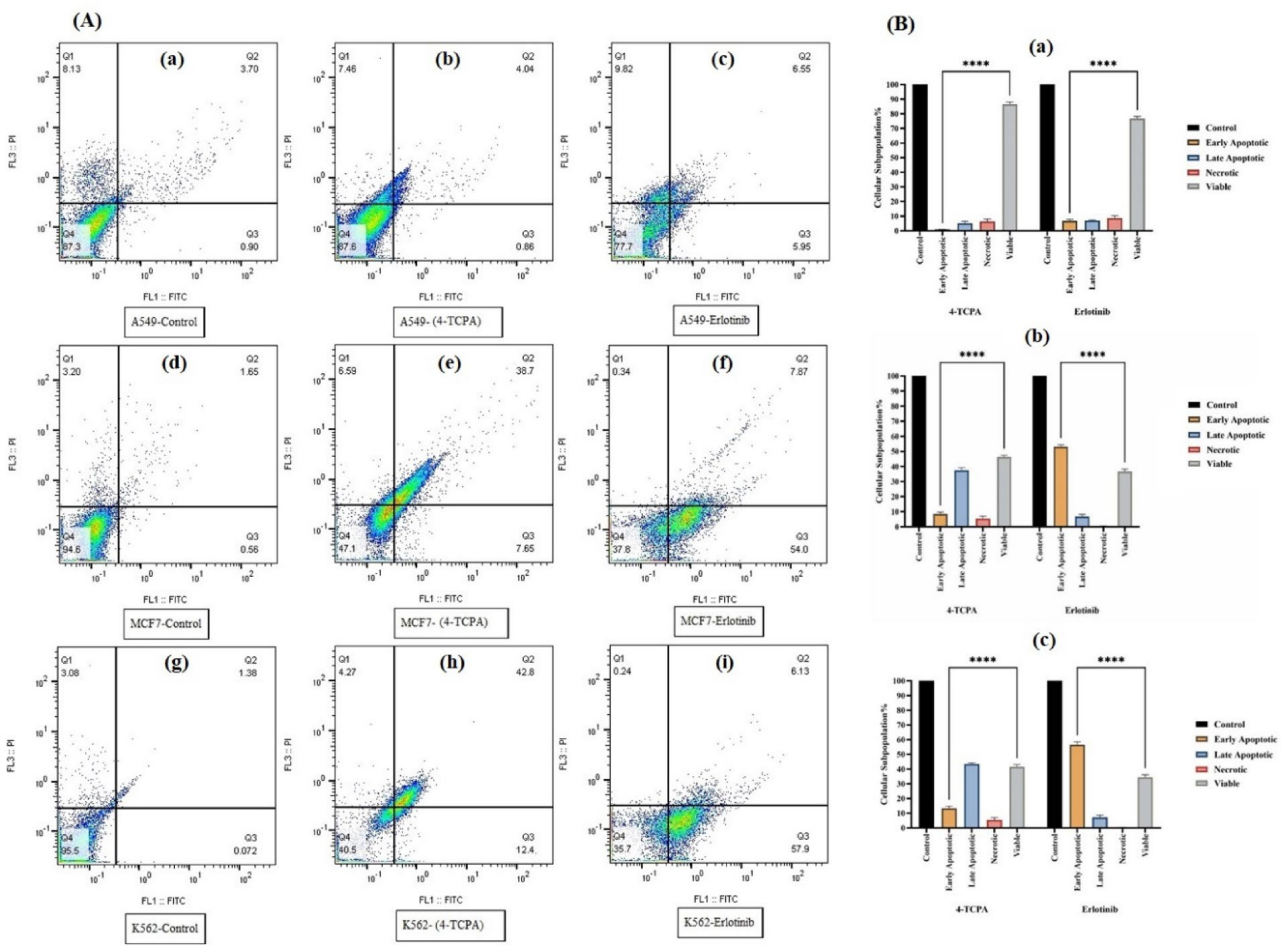
A549, MCF7, and K562 cells treated with **4-TCPA** and Erlotinib (as a positive control) were subjected to a quantitative annexin V/PI double staining test to measure apoptosis. (**A**) **4-TCPA** caused apoptosis in A549 (a,b,c), MCF7 (d,e,f), and K562 (g,h,i) cells in a dose- and time-dependent manner, according to flow cytometric analysis in. (**B**) The graph shows the statistical quantification of data A549 (a), MCF7 (b) and K562 (c) following **4-TCPA** treatment. Apoptosis analysis using flow cytometry revealed a time- and dose-dependent increase in early and late apoptotic cells. The data reflect the mean plus standard deviation (SD) of three separate studies. Comparing each group to its control resulted in ***p < ****p < 0.0001.

As directed by the manufacturer, annexin V/PI double labeling was used to measure the apoptotic rate of A549, MCF7, and K562 cells. After the cells were exposed to the compound’s IC_50_ concentration, flow cytometry was used to look for signs of apoptosis. The results showed that early and late apoptosis in all cell lines increased significantly with time (Fig. 3A). When all cells were treated with **4-TCPA**, after 72 h, more than 40% of the treated cells underwent apoptosis in contrast to the untreated cells except A549 cell line that was less than 40% of the treated cells underwent apoptosis in contrast to the untreated cells (Fig. 3B). However, the A549, MCF7 and K562 cells exposed with Erlotinib (as a positive control) for 72 h, only shown a 12.5%, 61.87% and 64.03% increase in apoptosis, respectively (Fig. 3A,B).

### Activation of caspase 3/7

**Fig. 4:**
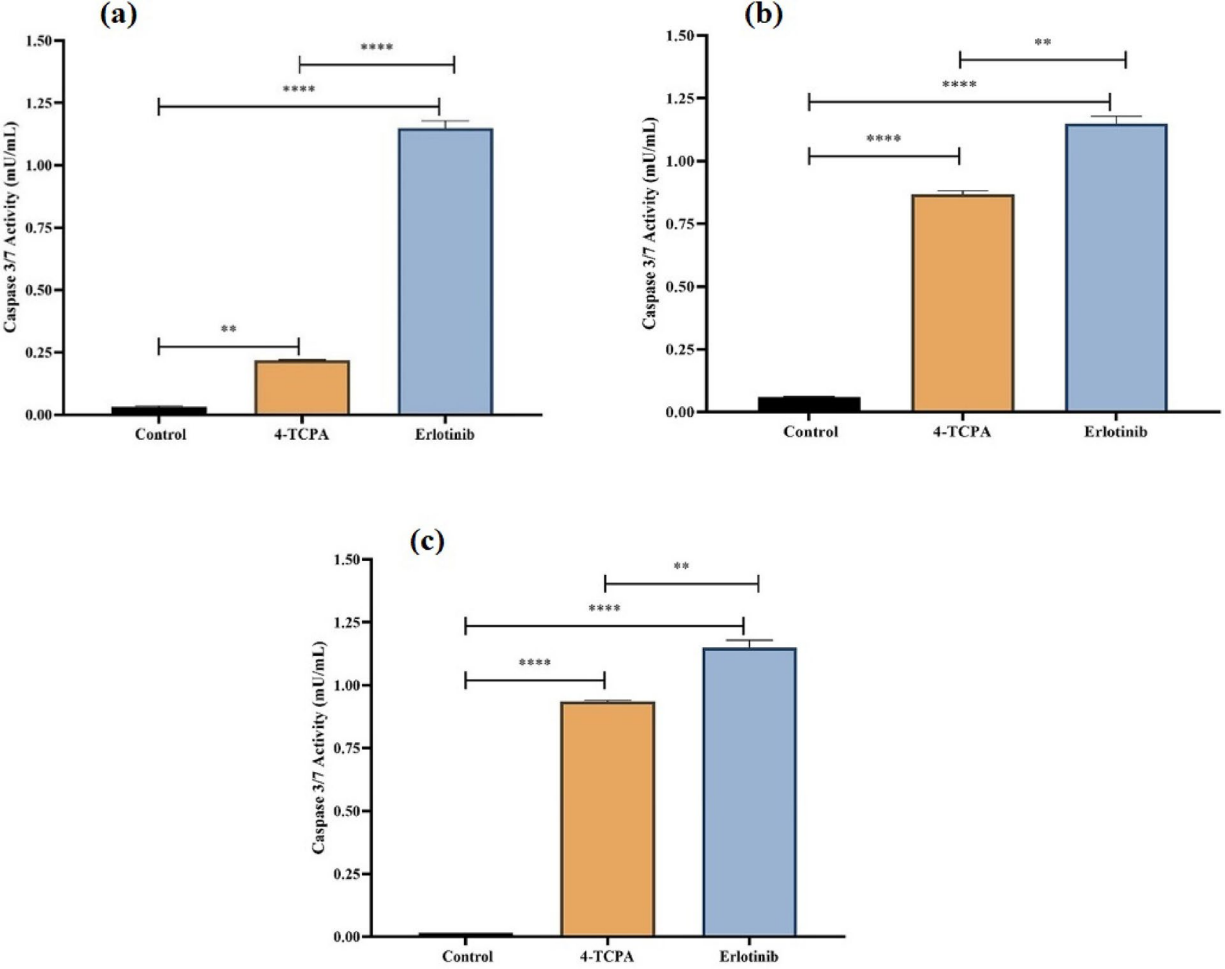
Assessment of caspase 3/7 enzymatic activities. The activation of caspase 3/7 was evaluated in A549 (**a**), MCF7 (**b**) and K562 (**c**) cell lines after exposure to the **4-TCPA** at indicated time point (72h), as explained in the material and method. Plots represent caspase 3/7 enzymatic activity (mU/mL) of the treated cells relative to the untreated groups and positive control (Erlotinib). The results are expressed as mean ± SD (*n* = 3) (****p < 0.0001 (A549), **p < 0.0013 (MCF7), **p < 0.0023 (K562)) versus positive control.

Using spectrophotometry, the enzymatic activity of caspase 3/7 were evaluated as markers of apoptosis in order to verify the existence of apoptosis. Compared to the untreated control cells and positive control (Erlotinib), **4-TCPA** treatment (at IC_50_ concentrations) consistently increased the activity of caspase 3/7 in a time-dependent manner in A549, MCF7, and K562 cells. After 72 h of treatment, this increase was 0.21 mU/mL for A549 cells (Fig. 4a), 0.86 mU/mL for MCF7 cells (Fig. 4b), and 0.93 mU/mL for K562 cells (Fig. 4c), respectively. These results corroborated the findings of qualitative and quantitative apoptosis investigations, which demonstrated that cancer cells enhanced apoptosis more and earlier.

### Evaluation of gene expression levels

**Fig. 5:**
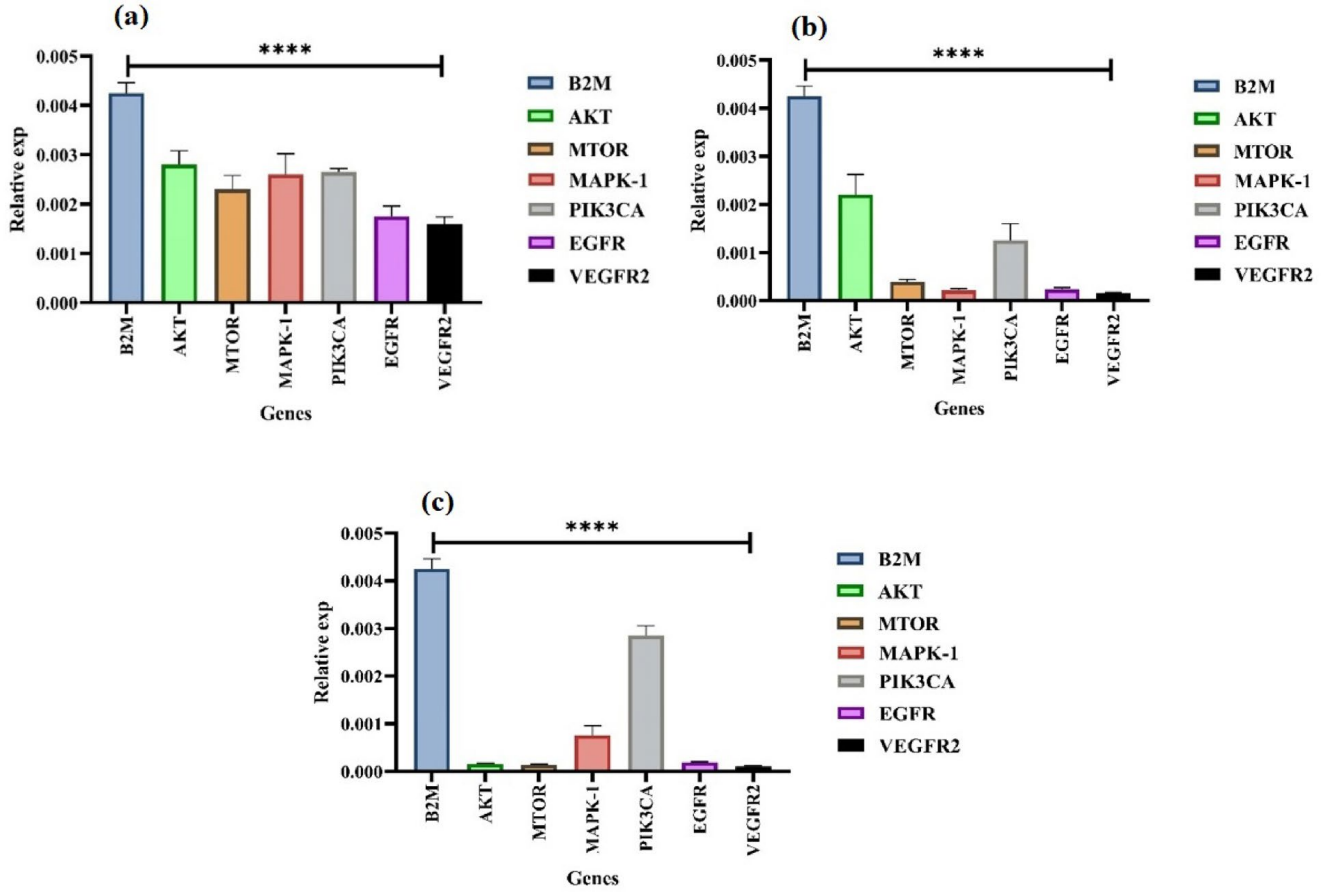
Measurement of mRNA expression levels following **4-TCPA** exposure. At the designated time, **4-TCPA** with an IC_50_ concentration was treated with A549 (**a**), MCF7 (**b**), K562 (**c**), cells. qRT-PCR was used to assess changes in the transcript levels of Akt, mTOR, MAPK, PIK3CA, EGFR and VEGFR2. Plots represent Akt, mTOR, MAPK, PIK3CA, EGFR and VEGFR2 expression levels of the treated cells relative to the control (B2M). The findings are presented as the mean SD (n = 3); (****p < 0.0001 (A549), (MCF7) and (K562) VEGFR2) versus control.

Using qRTPCR, the expression levels of the genes Akt, mTOR, MAPK-1, PIK3CA, EGFR, and VEGFR2 were determined to identify the processes by which mitochondria are implicated in apoptosis. After 72 h of treatment in A549, MCF7 and K562 cells, the results showed that the IC_50_ concentration of **4-TCPA** induced a significant down-regulation in the mRNA levels, respectively (Fig. 5). The outcome showed that A549 cell line had considerably lower levels of EGFR and VEGFR2 expression also lower levels of mTOR expression, but not as much as EGFR and VEGFR2 compared to the control group. Akt, MAPK-1, and PIK3CA expression levels did not decrease as much as those of other genes compared to the control group (Fig. 5a). The MCF7 cell line had considerably lower levels of mTOR, MAPK-1, EGFR and VEGFR2 expression also lower levels of PIK3CA expression, but not as much as EGFR and VEGFR2 compared to control group. The expression level of Akt was not decreased as much as other genes compared to the control group (Fig. 5b). The K562 cell line had considerably lower levels of Akt, mTOR, EGFR, and VEGFR2 expression also lower levels of MAPK-1 expression, but not as much as EGFR and VEGFR2 compared to the control group. The expression level of PIK3CA was not decreased as much as other genes compared to control group (Fig. 5c). These findings demonstrate that **4-TCPA** activated the mitochondrial pathway with varying sensitivity, causing apoptotic cell death in all cell lines. These results validated the qRT-PCR findings in A549, MCF7, and K562 cells.

## Discussion

Many cancer types that are in advanced stages of development are still incurable, even with the increasing availability of new anti-cancer drugs and sophisticated treatment regimens. Certain chemotherapy drugs target cancer cells but leave cancer stem cells (CSCs) unharmed, which increases the risk of disease recurrence and treatment resistance^31^. As a result, one characteristic of cancer is altered kinase and phosphatase functioning. Although kinases and phosphatases have been extensively studied about cancer, their abnormal activation within the CSC compartment is currently being researched as a potential new Achille’s heel. Cancer is believed to result from mutations in kinases or phosphatases that induce either a gain or a loss of function. Furthermore, oncogenes can alter the ratio of kinase to phosphatase activity, leading to malignant transformation of cells^4^. In cancer, it has often been found that unchecked kinase activation and phosphatase repression increase cell proliferation, migration, and survival in response to anti-cancer therapy. Failure to maintain the proper balance between kinases and phosphatases activity has been linked to multiple solid cancer forms, including breast, gastric, liver, and colorectal cancer(BC)^32^. Therefore, it stands to reason that a deeper comprehension of the roles and regulation of kinases and phosphatases will facilitate the discovery of novel anticancer drugs. A tiny subset of cancer cells known as cancer stem cells is thought to be responsible for cancer recurrence and relapse^33^. In addition, CSCs can begin and promote tumorigenesis and the creation of metastases. They can also engage in self-renewal and multilineage differentiation^34^. Both phosphatases and kinases are vital components of the cell’s regulatory network and a wide range of biological processes. While phosphatases eliminate phosphate groups from their target proteins, kinases facilitate the transfer of phosphate groups—which are liberated by ATP—to molecules. By altering a protein’s activity, stabilizing it, designating it for destruction, localizing it inside a specific cellular compartment, and causing or impairing its interaction with other proteins, kinase phosphorylation can change a protein’s function. Different proteins can act as substrates for more than one particular kinase, and protein kinases frequently work on several substrates. As a result of mediating most of a cell’s signal transmission, kinases regulate a wide range of cellular functions, including transcription, growth, death, metabolism, interaction with immune systems, migration, cytoskeletal reorganization, and differentiation. Protein kinases, which phosphorylate proteins at serine/threonine, tyrosine, or all three residues (dual-specificity kinases), comprise the most significant class of kinases^4^. The vascular endothelial growth factor receptor 2 (VEGFR2) is a pivotal target in cancer therapy due to its role in tumor angiogenesis^35^. Recent studies have highlighted the importance of inhibiting VEGFR2 to disrupt the blood supply to tumors, thereby limiting their growth and metastasis^36^. The novel quinazoline-containing 1,2,3-triazole compound **(4-TCPA)**, demonstrates promising potential as a VEGFR2 inhibitor, aligning with current therapeutic strategies to target angiogenesis. This study investigated the synthesis and anti-cancer activity of **4-TCPA**, focusing on its potential as VEGFR2 inhibitors. The results demonstrated that **4-TCPA** exhibited the potent cytotoxic effects against multiple cancer cell lines, inducing apoptosis and cell cycle arrest through modulation of key signaling pathways. The cytotoxicity assays revealed that **4-TCPA** had superior activity compared to the positive control erlotinib across the tested cancer cell lines. Results indicated broad-spectrum anti-cancer potential, particularly effective against the K562 leukemia cells (IC_50_ 5.95 μM). Notably, **4-TCPA** showed reduced cytotoxicity (IC_50_ 135.2 μM) against the normal HFF2 fibroblast cells, suggesting some degree of cancer cell selectivity. The structure–activity relationship analysis suggests the 1,2,3-triazole moiety contributes significantly to the anti-proliferative effects, while the azide substituent may modulate activity. Flow cytometry analysis showed that **4-TCPA** induces apoptosis compared to untreated controls and erlotinib in the cancer cell lines. Annexin V/PI staining quantified increasing early and late apoptotic cell populations in a time-dependent manner across all three cancer lines. The activation of caspase 3/7 further confirmed this pro-apoptotic effect, a critical executioner of programmed cell death. The caspase activity increased significantly after 72 h of **4-TCPA** treatment compared to both untreated controls and erlotinib. This study examined the expression of several genes involved in key oncogenic signaling pathways to elucidate potential molecular mechanisms. qRT-PCR analysis revealed that **4-TCPA** treatment led to significant downregulation of VEGFR2 and EGFR across all three cancer cell lines. This supports the hypothesis that the compound may act as a VEGFR2 inhibitor, interfering with angiogenesis signaling. The downregulation of EGFR is also noteworthy, as it is a major driver of proliferation in many cancers. Interestingly, the effects on other signaling molecules varied between cell lines. In A549 cells, mTOR was moderately downregulated, while Akt, MAPK, and PIK3CA showed minimal changes. MCF7 cells exhibited strong downregulation of mTOR, and MAPK, and moderate reduction in PIK3CA expression. K562 cells showed significant decreases in Akt and mTOR expression. These differential effects may reflect the heterogeneity of signaling pathway dependencies across cancer types. The consistent downregulation of VEGFR2 and EGFR across all lines suggests these may be primary targets of **4-TCPA**, with secondary effects on downstream pathways varying by cellular context. The observed downregulation of mTOR is particularly interesting, as the mTOR pathway integrates signals from multiple oncogenic inputs to regulate cell growth, proliferation, and survival. Inhibition of mTOR signaling has been shown to sensitize cancer cells to apoptosis and enhance the efficacy of other targeted therapies. The concurrent downregulation of VEGFR2, EGFR, and mTOR by **4-TCPA** may create a multi-pronged attack on cancer cell survival and proliferation pathways. While these results are promising, several limitations and areas for future research should be noted. First, the study focused on in vitro effects in cancer cell lines. Further investigation in animal models will be crucial to assess the in vivo efficacy, pharmacokinetics, and potential toxicities of **4-TCPA**. Additionally, while gene expression changes were observed, protein-level validation through Western blotting or other techniques would strengthen the mechanistic conclusions. Direct binding assays with purified VEGFR2 protein could confirm if **4-TCPA** acts as a direct kinase inhibitor. The differential effects observed across cancer cell lines highlight the importance of understanding the molecular context in which novel compounds act. Future studies could explore the efficacy of **4-TCPA** in a broader panel of cancer cell lines with defined genetic backgrounds. This could help identify biomarkers that predict response and guide potential clinical applications. Mechanistic investigations into **4-TCPA** reveal its ability to induce apoptosis and cell cycle arrest in cancer cell lines, a critical aspect of its anti-cancer activity. The downregulation of VEGFR2 and EGFR expression suggests that **4-TCPA** may effectively interfere with key signaling pathways involved in cancer progression. This multi-target approach could enhance the therapeutic efficacy of **4-TCPA**, making it a valuable candidate for further development. Moreover, the modulation of other signaling molecules, such as mTOR, Akt, and MAPK by **4-TCPA** indicates a synergistic effect that could be leveraged in combination therapies. Recent literature supports the notion that targeting multiple pathways can improve cancer treatment outcomes. This highlights the potential of **4-TCPA** not only as a standalone therapy but also in combination with existing treatments. Future studies should focus on in vivo models to validate the efficacy and safety of **4-TCPA**. The promising in vitro results warrant further exploration of its pharmacokinetics and pharmacodynamics in a clinical setting. Understanding the compound’s behavior in a biological system will be crucial for its advancement towards clinical application. Combination studies with existing therapies would also be valuable. Given the observed effects on multiple signaling pathways, **4-TCPA** might synergize with other targeted agents or chemotherapies. For example, the downregulation of mTOR could potentially sensitize cells to PI3K inhibitors. From a medicinal chemistry perspective, further structure–activity relationship studies could optimize the potency and selectivity of the compound. The current study identified the importance of the 1,2,3-triazole moiety, but a systematic modification of other molecule regions could yield derivatives with improved pharmacological properties.

## Conclusion

In conclusion, this study has identified a novel quinazoline-containing 1,2,3-triazole compound with promising anti-cancer activity across multiple cell lines. **4-TCPA** induces apoptosis and cell cycle arrest, likely through inhibition of VEGFR2 and EGFR signaling with additional effects on the mTOR pathway. These findings provide a strong rationale for further developing this compound class as potential cancer therapeutics. Future in vivo studies and mechanism elucidation will be crucial next steps in advancing this promising lead toward potential clinical application.

## Electronic supplementary material

Below is the link to the electronic supplementary material.

Supplementary Material 1

## Data Availability

All data generated or analyzed during this study are within the article.
