# Supplementary material for: Synthesis, characterization, anti-proliferative, and apoptotic activity of a novel quinazoline-containing 1,2,3-triazole toward three human cancer cells
Source: Sci Rep. 2025 Jul 1;15:20852. doi: 10.1038/s41598-025-05270-z (PMC12214827; doi:10.1038/s41598-025-05270-z)
Supplement: Supplementary file 1 — Supplementary Material 1 [file 41598_2025_5270_MOESM1_ESM.docx]

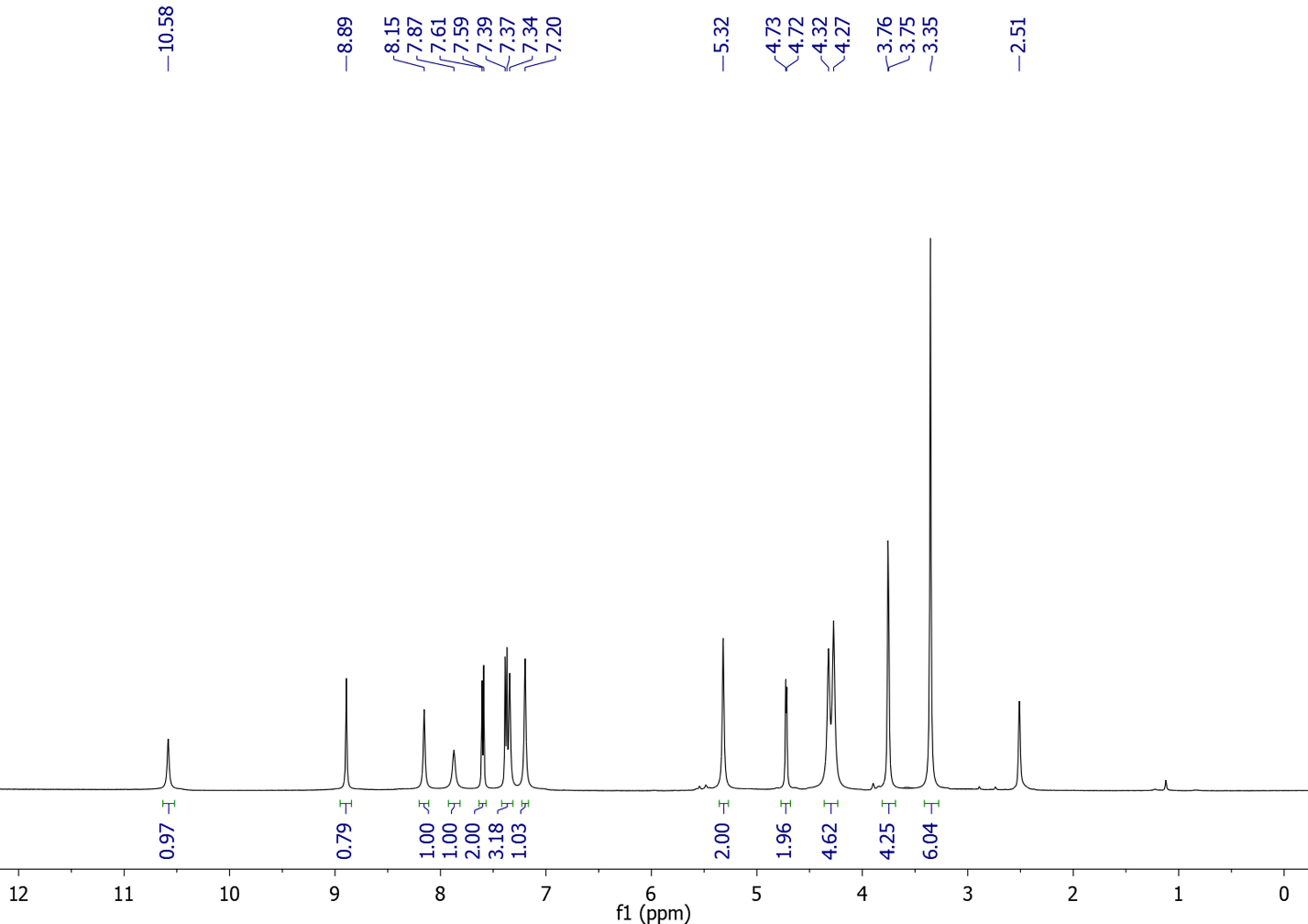


**Figure S1.** ^1^H-NMR spectrum of the novel quinazoline-containing 1,2,3-triazole compound (4-TCPA).


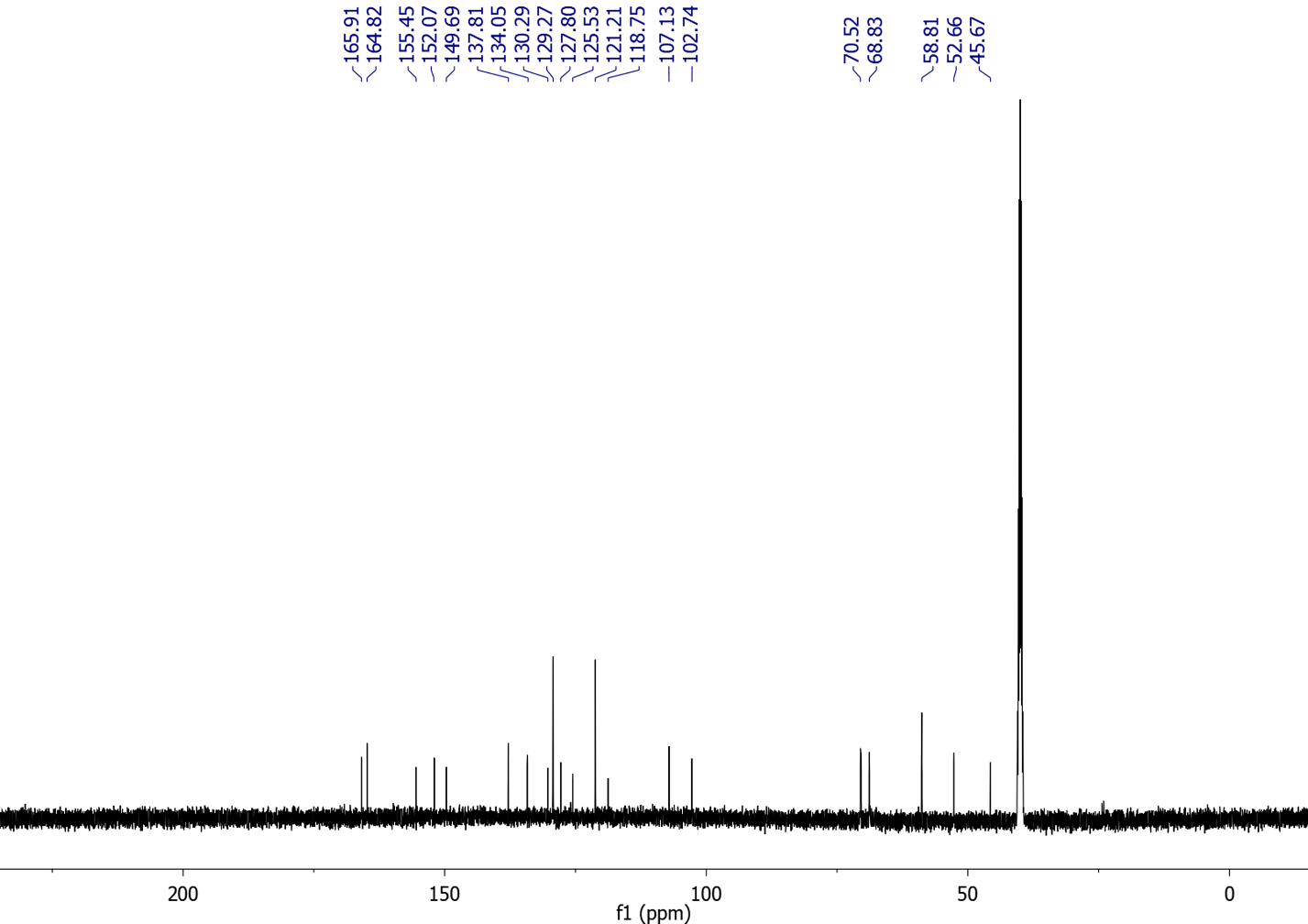


**Figure S2.** ^13^C-NMR spectrum of the novel quinazoline-containing 1,2,3-triazole compound (4-TCPA).

**
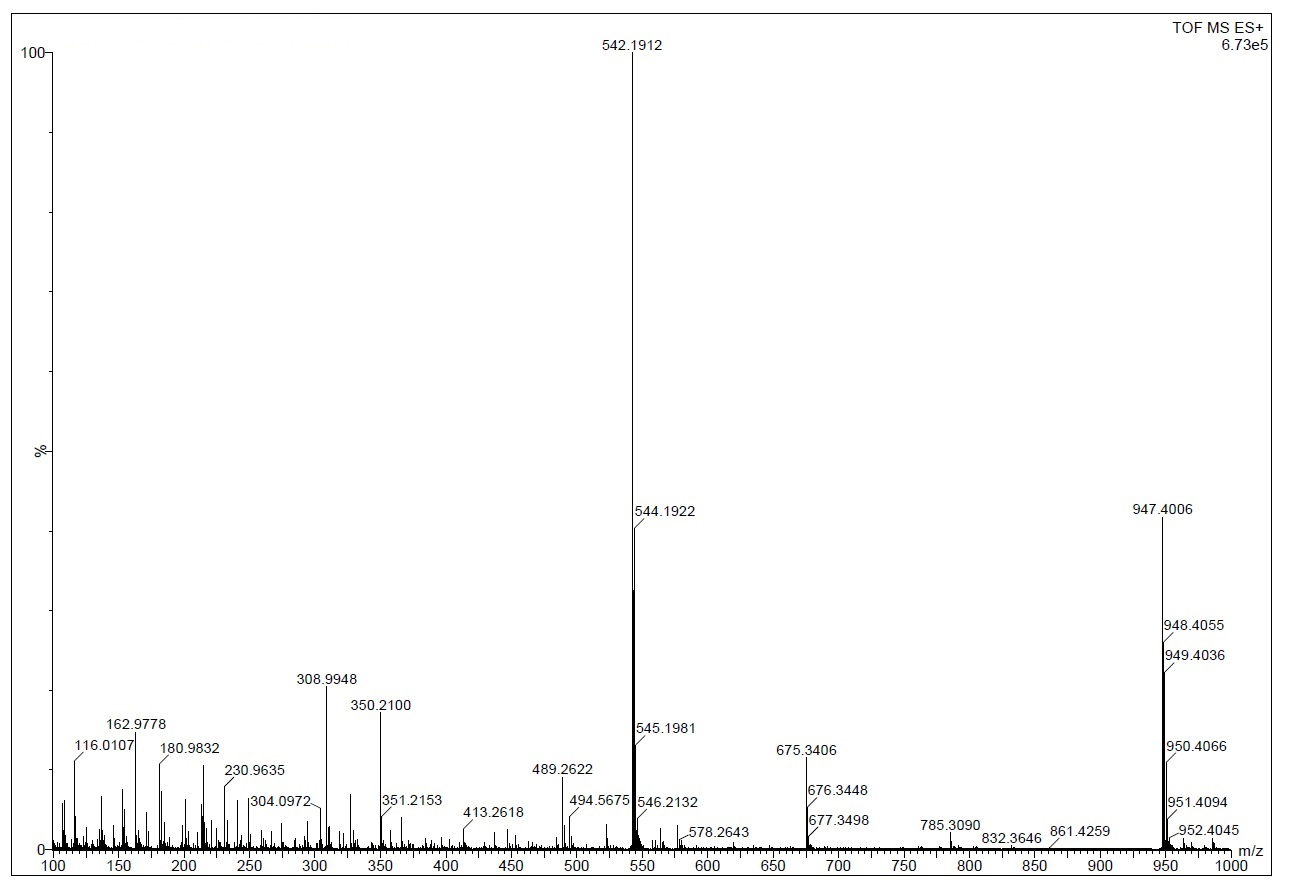
**

**Figure S3.** high-resolution mass spectrometry (HRMS) of the novel quinazoline-containing 1,2,3-triazole compound (4-TCPA).
